# Multicentric and Multifocal Breast Tumors—Narrative Literature Review

**DOI:** 10.3390/cancers17203380

**Published:** 2025-10-20

**Authors:** Mircea-Octavian Poenaru, Mihaela Amza, Cristian-Valentin Toma, Fernanda-Ecaterina Augustin, Irina Pacu, Giorgia Zampieri, Liana Ples, Romina-Marina Sima, Andrei-Sebastian Diaconescu

**Affiliations:** 1 Department of Obstetrics and Gynecology, “Carol Davila” University of Medicine and Pharmacy, 020021 Bucharest, Romania; mircea.poenaru@umfcd.ro (M.-O.P.); mihaela.amza@umfcd.ro (M.A.); irina.pacu@umfcd.ro (I.P.); giorgia.zampieri@drd.umfcd.ro (G.Z.); liana.ples@umfcd.ro (L.P.); romina.sima@umfcd.ro (R.-M.S.); 2 “Bucur” Maternity, Saint John Hospital, 012361 Bucharest, Romania; 3 Department of Urology, “Carol Davila” University of Medicine and Pharmacy, 020021 Bucharest, Romania; 4 “Prof. Dr. Theodol Burghele” Clinical Hospital, 061344 Bucharest, Romania; 5 “St. Pantelimon” Emergency Clinical Hospital, 021623 Bucharest, Romania; 6 Department of General Surgery, Faculty of Medicine, “Carol Davila” University of Medicine and Pharmacy, 050474 Bucharest, Romania; andrei.diaconescu@umfcd.ro; 7 General Surgery Department, Fundeni Clinical Institute, 022328 Bucharest, Romania

**Keywords:** multifocal breast cancer, multicentric breast cancer, multiple ipsilateral breast tumors, breast-conserving surgery, prognosis

## Abstract

**Simple Summary:**

Multifocal and multicentric breast cancers refer to the presence of two or more distinct tumor foci within the same breast. These forms present diagnostic, surgical, and prognostic challenges, as multiple lesions may differ in biology and therapeutic response. Historically, such cases were considered contraindications for breast-conserving surgery due to concerns of incomplete excision and higher recurrence risk. Recent advances in imaging, oncoplastic techniques, and systemic therapies have changed clinical practice. This narrative review synthesizes current evidence regarding incidence, pathological characteristics, imaging modalities, treatment strategies, and patient outcomes for multifocal and multicentric breast tumors. By integrating data from more than fifty recent studies, the review highlights that, with accurate staging and multidisciplinary management, survival outcomes can approach those of unifocal disease. These insights contribute to refining surgical decision-making and support a more individualized approach to breast cancer treatment.

**Abstract:**

Background: Multifocal (MF) and multicentric (MC) breast cancers, defined by the presence of multiple synchronous tumor foci within the same breast, present important diagnostic, therapeutic, and prognostic challenges. Historically considered a contraindication for breast-conserving therapy (BCT), advances in imaging, surgical techniques, and adjuvant therapy have reshaped management strategies. Methods: A narrative literature review was conducted through PubMed, Web of Science, and Scopus, prioritizing ISI-indexed articles published within the last 10–15 years. More than 55 relevant studies, including systematic reviews, meta-analyses, and large cohorts, were analyzed to evaluate epidemiology, pathological features, imaging modalities, treatment outcomes, and prognosis of MF/MC breast cancers. Results: The reported incidence of MF/MC breast cancers ranges from 10% to 24%, increasing when MRI or whole-organ pathology is applied. MRI can detect otherwise occult additional foci in up to 30% of patients, improving staging accuracy but raising concerns of overdiagnosis. MF/MC presentation is strongly associated with lobular histology, younger age at diagnosis, and higher rates of axillary involvement—nodal positivity is observed in up to 45% of MF/MC cases versus 28% in unifocal tumors. Pathological analyses demonstrate frequent clonal origin of MF lesions, whereas MC lesions may represent independent primaries, occasionally with receptor heterogeneity that alters systemic therapy selection. From a prognostic perspective, older series suggested shorter breast cancer-specific survival (e.g., median 154 vs. 204 months for MF/MC vs. unifocal disease), and higher local recurrence with BCT. However, contemporary analyses, including a 2022 meta-analysis of 15,703 patients, demonstrated no significant difference in overall or disease-free survival once adjusted for tumor size and nodal status. Local recurrence remains slightly higher with BCT in MF/MC (5.6% vs. 4.2%), but outcomes are equivalent to mastectomy when radiotherapy is appropriately delivered. Five-year survival in early-stage MF/MC exceeds 90% with guideline-concordant multimodal therapy. Conclusions: MF/MC breast cancers represent a biologically heterogeneous entity. Optimal outcomes rely on precise imaging, complete excision, tailored systemic therapy, and multidisciplinary management, with increasing acceptance of breast conservation in selected patients.

## 1. Introduction

Multiple breast tumors represent a significant challenge in breast oncology, with profound implications for diagnosis, treatment, and patient prognosis. This phenomenon, characterized by the presence of two or more distinct tumors within the same breast or both breasts, holds major relevance from various clinical and research perspectives. Although the presence of multiple lesions has historically been considered a risk factor for poor prognosis, recent studies advocate for a more nuanced approach based on the molecular and histological heterogeneity between lesions.

Breast malignancies can manifest as many synchronous tumor foci within the same breast, referred to as multifocal or multicentric tumors. Multifocal (MF) illness denotes the presence of two or more separate tumor foci situated inside the same quadrant or section of the breast, generally within 2–5 cm of one another [1,2].

On the other hand, multicentric (MC) illness comprises numerous tumors situated in distinct quadrants or regions of the breast, typically separated by more than 5 cm or located within different breast lobes [1,2]. The distinction is often nuanced, since some clinicians categorize both as “multiple ipsilateral breast cancers” due to difficulties in delineating quadrant borders [3]. Nonetheless, MF tumors are typically regarded as clonally linked lesions within the same breast sector, whereas MC tumors may signify independent original foci in other sectors [4]. The quadrant-based classification is criticized as anatomically arbitrary, not reflecting lymphatic drainage [5].

Quadrant-based definitions of multifocality/multicentricity remain descriptive for imaging and operative planning but have limited prognostic or therapeutic value in the molecular era. Current staging assigns T by the **largest invasive focus with an “(m)” suffix**, independent of quadrant boundaries, reflecting the primacy of tumor biology and nodal status. Consensus guidance likewise advocates biology-driven management and selective MRI use (e.g., dense breasts), rather than quadrant location, to refine extent assessment and treatment planning. Accordingly, we report multiple ipsilateral breast cancers by number of foci, inter-lesional distance, and receptor/genomic concordance, reserving quadrant terminology for anatomic clarity [6]

The clinical significance of multifocality and multicentricity in breast cancer is substantial. Historically, the existence of several tumor foci was considered a contraindication for breast-conserving therapy, due to concerns regarding residual disease and recurrence [7].

Consequently, numerous patients of this kind have been treated with mastectomy. However, advancements in imaging, surgical techniques, and adjuvant therapies have increased interest in the viability of breast-conserving surgery (BCS) for multifocal/multicentric illness and in understanding the prognostic impact of multifocality [8].

Table 1 summarizes the differences in incidence, imaging characteristics, surgical and systemic treatment approaches, histopathological findings, and prognosis between multifocal (MF), multicentric (MC), and unifocal breast cancer.

This review aims to delineate multifocal from multicentric breast cancer, synthesize epidemiological data and risk factors, elucidate the distinctive pathological and biological characteristics of MF/MC tumors, examine diagnostic and imaging methodologies, assess contemporary clinical management and treatment strategies, analyze the influence of multifocality/multicentricity on prognosis, and underscore existing controversies and prospective research possibilities in this domain.

Unlike prior syntheses focused primarily on the feasibility of breast conservation and historical outcomes in MF/MC disease, our review integrates post-2019 advances that reshape practice: ultra-hypofractionated adjuvant radiotherapy (FAST-Forward), contemporary oncoplastic strategies extending conservation to complex MF/MC anatomies, and genomic evidence of inter-lesional heterogeneity informing systemic therapy choices. We also review prognosis based on recent meta-analyses, separating local control signals from stage-adjusted survival.

## 2. Materials and Methods

A structured literature search was performed between January 2010 and September 2024 using PubMed, Scopus, and Web of Science databases. The search terms included combinations of “multifocal breast cancer,” “multicentric breast cancer,” “multiple ipsilateral breast tumors,” “oncoplastic surgery,” “prognosis,” “molecular heterogeneity,” and “radiotherapy.” Inclusion criteria prioritized peer-reviewed articles with an impact factor of at least 2 from ISI-indexed journals, focusing on studies reporting incidence, risk factors, pathological characteristics, imaging modalities, treatment outcomes, or prognostic data specific to multifocal/multicentric breast cancers. Classic studies were also considered for historical context. Exclusion criteria involved non-peer-reviewed publications, studies lacking sufficient clinical or pathological detail, or those outside the defined timeframe, ensuring the collection of robust and clinically relevant data. The exclusion criteria also covered case reports, small series with fewer than 20 patients, non-English publications, and conference abstracts. The initial search yielded 512 records, of which 82 full texts were screened; more than 75 articles met the inclusion criteria and were analyzed in this narrative synthesis. Reference lists of relevant reviews and meta-analyses were cross-checked to identify additional eligible studies. No restriction on study design was applied beyond clinical relevance.

**Figure 1:**
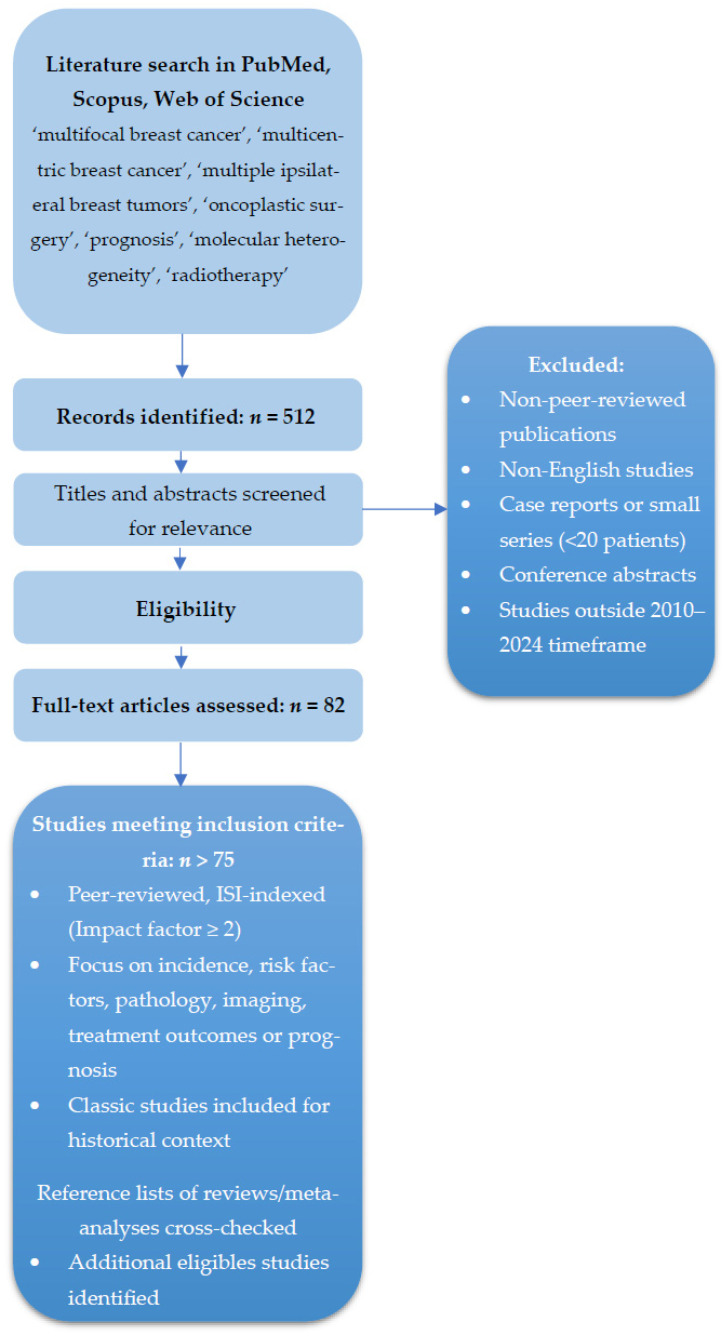
Flowchart illustrating the literature selection process for the narrative review on multifocal and multicentric breast cancer. The diagram outlines the identification, screening, eligibility, and inclusion stages, summarizing the search strategy, exclusion criteria, and final study selection.

As this study was designed as a narrative rather than a systematic review, some degree of selection bias cannot be entirely excluded. Nevertheless, this limitation was mitigated by predefined eligibility criteria, independent dual screening of retrieved records, and prioritization of ISI-indexed studies with high methodological quality. Using multiple databases and manually checking references improved coverage and minimized bias in selecting literature (Figure 1).

As this study was a literature review without new human subject research, no institutional review board approval was required.

## 3. Epidemiological Aspects

### 3.1. Incidence

The documented prevalence of multifocal/multicentric breast cancer exhibits considerable variability in the literature, primarily because of discrepancies in detection methodologies and terminology [8]. Previous research indicated incidence rates varying from approximately 6% to 60%, with elevated rates observed in studies employing comprehensive pathological analysis of mastectomy specimens [9]. In modern clinical studies, MF/MC tumors are identified in approximately 10–20% of breast cancer patients. A substantial single-institution study from Italy indicated that MF cancer was present in 11.3% and MC in 5.2% of 1,158 breast cancer cases [1]. A meta-analysis of over 19,000 individuals revealed that around 6.2% were diagnosed with multifocal or multicentric illness [10]. Contemporary large-scale analyses indicate that MF/MC tumors are identified in approximately 10–24% of all breast cancers, with higher detection rates in studies employing MRI or whole-organ pathological examination [1]. This consolidated range aligns with the recent meta-analysis by Zhang et al. (2022), encompassing over 240,000 cases and demonstrating that incidence increases proportionally with imaging sensitivity and pathological sampling depth [11,12].

Apparent geographic disparities in the incidence of multifocal and multicentric breast cancer primarily arise from methodological and diagnostic heterogeneity rather than intrinsic biological differences. Lower rates reported in Asian populations (1–5%) compared with Western series (10–20%) correlate strongly with differences in screening coverage, MRI utilization, and pathological sampling protocols [3]. In countries with widespread MRI use, detection of additional foci increases by up to 30%. Nonetheless, biological and demographic contributors—including smaller average breast volume, higher tissue density, and the lower prevalence of lobular carcinoma—may modestly influence these patterns [8]. Overall, the data support that improved imaging sensitivity, rather than regional tumor biology, explains most intercontinental variability.

### 3.2. Associated Risk Factors

There is no single known risk factor unique to multifocal or multicentric breast cancer, but certain tumor and patient characteristics have been associated with MF/MC presentations. Patients with MF/MC disease tend to have larger primary tumors and higher tumor burden. By definition, having multiple foci often implies a greater aggregate tumor volume even if the largest focus is of similar size to unifocal cancers [12]. For instance, one study noted the mean diameter of the dominant tumor was larger in multifocal cases (3.68 cm) than in unifocal cases (3.21 cm) [13]. Consequently, axillary lymph node involvement is more frequent—studies have shown lymph node positivity rates around 55–60% in MF/MC cases vs. ~45% in unifocal cases [3]. An earlier age at onset has been seen; regarding age group distribution, most studies report a higher prevalence of multifocal tumors in women aged between 45 and 65 years, with a peak incidence around the age of 55 [14]. Certain research indicates that endogenous hormonal factors may be influential; for instance, a specific case–control study discovered that patients with multifocal cancer exhibited a marginally younger average age at menarche compared to those with unifocal cancer (mean menarche ~13.0 years MF vs. 13.2 years UF, *p* = 0.03) [13]. The minor difference corroborates a prior study indicating that patients with early menarche and prolonged estrogen exposure may be vulnerable to multifocal tumors [13]. Reproductive factors also influence the risk of developing multiple breast tumors: women with fewer pregnancies and a later age at first childbirth are at increased risk. Nulliparity is a known risk factor frequently associated with multicentric tumors, particularly in lobular subtypes [15]. Additionally, histologic subtype is a notable factor—invasive lobular carcinoma (ILC) has a well-documented propensity for multiplicity. ILC tends to grow in a diffuse, infiltrative pattern and is frequently multifocal or multicentric (and bilateral) at presentation [16,17]. Studies indicate that 15–30% of ILC cases are multifocal or multicentric, a higher proportion than seen in the more common ductal carcinomas [18]. The loss of E-cadherin and discohesive growth in ILC likely contribute to this pattern, as microscopic foci can permeate the breast tissue. In contrast, high-grade ductal carcinomas or those with lymphovascular invasion may also present with satellite foci in the vicinity of the main tumor [16].

A family history of breast cancer, particularly in the presence of BRCA1 or BRCA2 mutations, is associated with an increased risk of developing multifocal or multicentric tumors. Some studies suggest that patients carrying BRCA mutations are more likely to present with synchronous bilateral and multifocal lesions [19].

Dense breast tissue can be a confounding factor. Women with radiologically dense breasts have cancers that are harder to find on mammograms, so a small multifocal tumor might not be found until surgery. In general, family history, reproductive factors, and other known breast cancer risk factors are the same for both MF/MC and unifocal cancers. However, lobular histology and factors related to tumor burden (size, grade) are more common in MF/MC cases [1].

### 3.3. Geographical and Demographic Trends

Regional variability exists in the reported incidence of multifocal/multicentric (MF/MC) breast cancer. Lower rates (1–5%) are observed in Asian populations (e.g., China, Korea), potentially due to differences in breast anatomy or diagnostic approaches [3]. The rising incidence of multifocal and multicentric breast cancer observed over the past decade largely reflects methodological improvements rather than a true biological increase. The widespread implementation of screening mammography, and particularly the use of breast MRI and contrast-enhanced mammography, has substantially enhanced the detection of additional tumor foci that were previously occult [18,20]. Advances in whole-organ pathological sectioning and improved histological mapping have further contributed to identifying multiple synchronous lesions. Consequently, reported rates vary across studies according to imaging modality, screening density, and pathology protocols rather than intrinsic regional or temporal differences [3,8].

In summary, the incidence of multicentric and multifocal breast tumors has increased over the past decade, largely due to advancements in imaging techniques—particularly the use of magnetic resonance imaging (MRI) and contrast-enhanced mammography [20]. Recent studies, conducted on exclusively British female cohorts, report a prevalence ranging from approximately 20% to 40% for multifocal and multicentric disease, with variability largely influenced by the diagnostic criteria and imaging modalities employed [21]. While demographic patterns exist, tumor biology and diagnostic protocols remain the primary determinants of multifocality.

## 4. Biological and Pathological Characteristics

Multifocal and multicentric breast tumors differ anatomically but share a common biological theme: the presence of multiple tumor foci within the breast. Multifocal tumors, located within the same quadrant, usually arise from local intramammary spread or simultaneous growths along the same ductal system. They tend to share histological and molecular features, effectively behaving as a single disease process with satellite lesions. In contrast, multicentric tumors, found in different quadrants, may either represent independent tumor clones or reflect spread through extensive ductal networks. Distinguishing between the two can be difficult in practice, as the definition of quadrants can be subjective—especially when tumor foci are only a few centimeters apart [22]. Multifocal disease is more commonly reported than true multicentric disease.

In some cases, what appears to be multicentric spread may actually be multifocal involvement that extends beyond quadrant borders. One study found that 74% of multiple tumors were multifocal, while only 26% were multicentric [23].

### 4.1. Nodal Involvement

Several studies have reported an association between MF/MC tumors and increased axillary lymph node involvement, indicative of a more aggressive tumor biology; however, when aggregate tumor burden metrics are considered, these differences may become less pronounced. In a prospective cohort study involving 812 patients, 141 with MF/MC tumors, tumor size—measured as largest focus diameter, aggregate diameter, or aggregate volume—was significantly associated with progression-free survival (PFS) and overall survival (OS). Among these metrics, the diameter of the largest tumor focus demonstrated the strongest prognostic value [24]. Supporting this statement is the study conducted by Wolters R on 8935 patients [25]. They found that for MF T1-T2 breast cancer, both breast-conserving therapy and mastectomy align with guidelines, while in MC cases, tumor size significantly affects survival [25].

### 4.2. Histological and Molecular Analysis

An important aspect of histopathological assessment is the identification of the tumor type present in each individual focus. Determining whether the foci are composed of the same histological subtype or represent distinct tumor entities has significant implications for staging, therapeutic planning, and prognostic evaluation. The most common subtypes include invasive ductal carcinoma (IDC), invasive lobular carcinoma (ILC), and, more rarely, medullary or papillary carcinoma. Although ILC is less frequent than IDC, it tends to exhibit a greater propensity for multifocality due to its diffusely infiltrative growth pattern. This biological behavior is associated with an increased risk of long-term recurrence, even after appropriate treatment. Another important aspect is the potential coexistence of non-invasive lesions, such as ductal carcinoma in situ (DCIS), alongside invasive components. Invasive ductal carcinoma is the most commonly encountered histological subtype, accounting for approximately 70–80% of cases of multifocal and multicentric breast tumors [26].

Pathologically, MF/MC tumors can be multifocal invasive ductal carcinomas, lobular carcinomas, or mixed. Often, the largest lesion (index tumor) dictates the tumor grade and receptor status reported, but careful analysis may find variation among foci. In most cases, the additional foci in multifocal breast cancer share the same histologic subtype as the primary [23]. However, in some multicentric cases, different foci can have different histologies—for example. One study found that about 20% of minor tumor foci were of a different pathological type than the main focus [23].

These observations are reinforced by earlier studies demonstrating that, in roughly 26.7% of ipsilateral multifocal breast cancer cases, discrepancies arise in surrogate molecular subtypes among the different tumor foci. Multifocal or diffusely distributed invasive breast tumors are associated with significantly worse outcomes compared to unifocal tumors. Specifically, patients with multifocal disease exhibit a 2.75-fold higher risk of breast cancer-related mortality, regardless of molecular subtype. This adverse prognostic impact remains significant even after multivariate adjustment for age, tumor stage, and nodal status. Importantly, the unfavorable prognosis linked to multifocality persists across different molecular phenotypes—including luminal A, HER2-positive, and basal-like subtypes—suggesting that the pattern of tumor distribution itself carries independent prognostic value [27]. This intertumoral heterogeneity carries major therapeutic implications, as it can influence the response to systemic treatment and the risk of local recurrence [28].

The immunohistochemical profile (IHC) of multicentric and multifocal breast tumors exhibits distinct characteristics compared to unifocal forms. Estrogen receptor (ER) and progesterone receptor (PR) expression show discordance in approximately 2.7% and 19.1% of tumor foci, respectively [27]. This variability in hormone receptor expression has direct implications for adjuvant hormonal therapy [21]. A recent study evaluated ER, PR, HER2, and Ki-67 in each focus of multifocal/multicentric cancers: approximately 24% of patients showed heterogeneity in molecular markers among different foci [23]. The most common discordance was in the Ki-67 proliferation index and hormone receptor status. Notably, 20% of MF/MC patients had different intrinsic molecular subtypes (Luminal vs. HER2-enriched vs. Basal) between different lesions. This intrabreast heterogeneity has clinical implications for the treatment (e.g., if one focus is HER2-positive and another is HER2-negative, clinicians typically treat as if the patient has HER2-positive disease to cover the higher-risk component. It also suggests that even clonally related foci can diverge in phenotype due to additional mutations or microenvironment influences in different parts of the breast [23].

Molecular profiling has clarified the origins of multiple tumor foci in the breast. Clonality studies—using methods such as X-chromosome inactivation patterns, loss of heterozygosity, and sequencing—suggest that most multifocal breast cancers arise from a single progenitor clone. For example, classic analyses show that approximately 90% of multifocal tumors share the same genetic or epigenetic markers, indicating a common origin, while about 10% appear genetically distinct, suggesting independent development. Pekár et al. (2014) similarly found that most foci in multifocal cancers share a “common ancestor” molecular signature, supporting the idea of local spread or intraductal extension from a single lesion [27]. In contrast, multicentric tumors—especially those located in different quadrants—are more likely to have independent clonal origins. Nonetheless, even spatially distant tumors can sometimes be clonally related [28]. Rare cases with distinct molecular subtypes within the same breast (e.g., one ER-positive, one ER-negative) clearly point to parallel primary tumors [23].

The emergence of multifocal or multicentric breast cancer is increasingly understood as a product of early clonal divergence and spatial dissemination. Genomic profiling across synchronous lesions often reveals discordant copy number alterations—for example, ERBB2, FGFR1, and FGFR2 amplifications variably present in distinct foci from the same patient—despite concordant driver mutations (e.g., PIK3CA) across lesions [29]. Single-cell and spatial transcriptomic studies further demonstrate differential subclonal architectures across adjacent foci, with divergent pathway activation (e.g., PI3K/AKT, DNA repair signatures). Jeon et al. (2022) documented variable immunophenotypes (ER, PR, HER2) even in contiguous invasive and DCIS lesions of multifocal breast cancer [30]. Such inter-lesional molecular heterogeneity may underlie variable treatment sensitivity and challenges in representative molecular sampling.

In summary, multifocal tumors usually represent a single clonal process with local dissemination, while multicentric tumors may reflect either clonal spread or independent tumorigenesis.

### 4.3. Biological Markers in Multifocal and Multicentric Breast Cancer

Traditional biomarkers—estrogen receptor (ER), progesterone receptor (PR), and HER2—retain their prognostic and predictive value in multifocal (MF) and multicentric (MC) breast cancers, as they do in unifocal cases. However, due to the presence of multiple malignant foci, thorough evaluation of all significant lesions is essential. Receptor expression is typically concordant across foci, but occasional discordance can occur, usually involving hormone receptor loss or HER2 gain in one focus—potentially reflecting clonal evolution [23].

In clinical practice, biomarker testing is often performed on the largest (index) tumor. Yet, if smaller foci show distinct histological features or appear more aggressive, separate testing is warranted—especially for lesions over 0.5 cm, since smaller foci may unexpectedly harbor HER2 amplification despite the main tumor being HER2-negative (or vice versa). From a research standpoint, differences in markers such as Ki-67 between foci may explain variations in proliferative behavior. Studies have also identified unique molecular patterns associated with multifocality. For instance, elevated levels of microRNA-429 in multifocal tumors suggest a potential “field effect” or inherent susceptibility within the breast tissue [31].

The “sick lobe hypothesis” further supports this idea, proposing that an entire breast lobe may undergo early, widespread pre-neoplastic changes (field cancerization), giving rise to multiple related tumor foci. This is consistent with findings that multifocal cancers often cluster within a single lobe and are associated with extensive ductal carcinoma in situ (DCIS) in the same region. Molecular profiling of DCIS and invasive components frequently confirms their clonal relationship [32].

In summary, while no single biomarker definitively predicts multifocality, features such as lobular histology, high proliferative index, and extensive DCIS are commonly associated. Ongoing genomic research may clarify why some tumors remain localized while others develop multiple foci.

Genomic heterogenity among tumor foci. A significant contribution to the understanding of multiple breast tumors comes from the study published in the British Journal of Cancer by Ahn et al. (2020) [29]. They performed genomic profiling of multiple lesions within the same patient and identified considerable heterogeneity, even in cases with similar histology. This finding underscores the importance of individual genomic testing for each tumor focus to enable personalized therapy. The results have direct implications for the validity of relying on a single biopsy for systemic treatment decisions [29].

These findings underscore that molecular divergence among tumor foci has direct implications for both staging and treatment. Therapeutic planning should therefore be guided by the most aggressive or biologically unfavorable lesion, reflecting current precision oncology principles. Incorporating molecular and clonal profiling into the evaluation of multifocal and multicentric breast cancers may refine prognostic assessment beyond traditional size-based criteria.

## 5. Diagnosis and Imaging

Accurate preoperative identification of multifocal or multicentric breast cancer is essential for guiding treatment. However, small secondary foci may go undetected with standard imaging, and distinguishing malignant from benign lesions remains challenging.

### 5.1. Imaging Techniques

#### 5.1.1. Mammography

Mammography may show multifocality via multiple clusters of microcalcifications or discrete masses, but it often underestimates disease extent, especially in dense breasts or without calcifications. Additional foci are frequently detected later through ultrasound (US), magnetic resonance imaging (MRI), or on final pathology. Key mammographic indicators of MF disease include segmental calcifications across a broad area or widely spaced lesions. However, lesions under 1 cm are often missed [1].

#### 5.1.2. Ultrasound

Breast ultrasound complements mammography and is especially helpful in dense tissue. Whole-breast US can reveal additional invasive foci not seen on mammograms, including those of invasive lobular carcinoma. Radiologists typically perform a quadrant-by-quadrant scan in patients with known tumors, detecting additional disease in roughly 10–15% of cases initially thought to be unifocal. While operator-dependent and prone to false positives, US is valuable for identifying nearby satellite nodules [33].

#### 5.1.3. Magnetic Resonance Imaging

MRI is the most sensitive tool for detecting MF/MC breast cancer. Contrast-enhanced MRI often reveals lesions missed by mammography and US, particularly in dense breasts. Studies show MRI can uncover additional foci in up to 16–20% of breast cancer cases, increasing mastectomy rates in institutions that routinely use it [34]. Most contemporary studies report that MRI identifies otherwise occult lesions in 10–30% of patients, depending on histology and breast density [20]. Higher detection rates have been observed in selected cohorts enriched with invasive lobular carcinoma. For instance, Milulescu et al. (2017) reported new MRI-detected lesions in 48.1% (39/81) of patients, though this study involved a small single-center cohort with specific inclusion criteria [35]. These findings highlight MRI’s diagnostic sensitivity but also underscore the need for histopathologic confirmation to avoid overestimation and overtreatment.

Given false-positive rates, biopsy confirmation of MRI-only lesions is essential to avoid unnecessary wider excisions/mastectomy. Still, its impact on long-term outcomes remains uncertain. MRI is particularly useful for invasive lobular carcinoma, inconclusive imaging, dense breasts, or when planning breast-conserving surgery [16].

### 5.2. Diagnostic Workup and Biopsy

**Figure 2:**
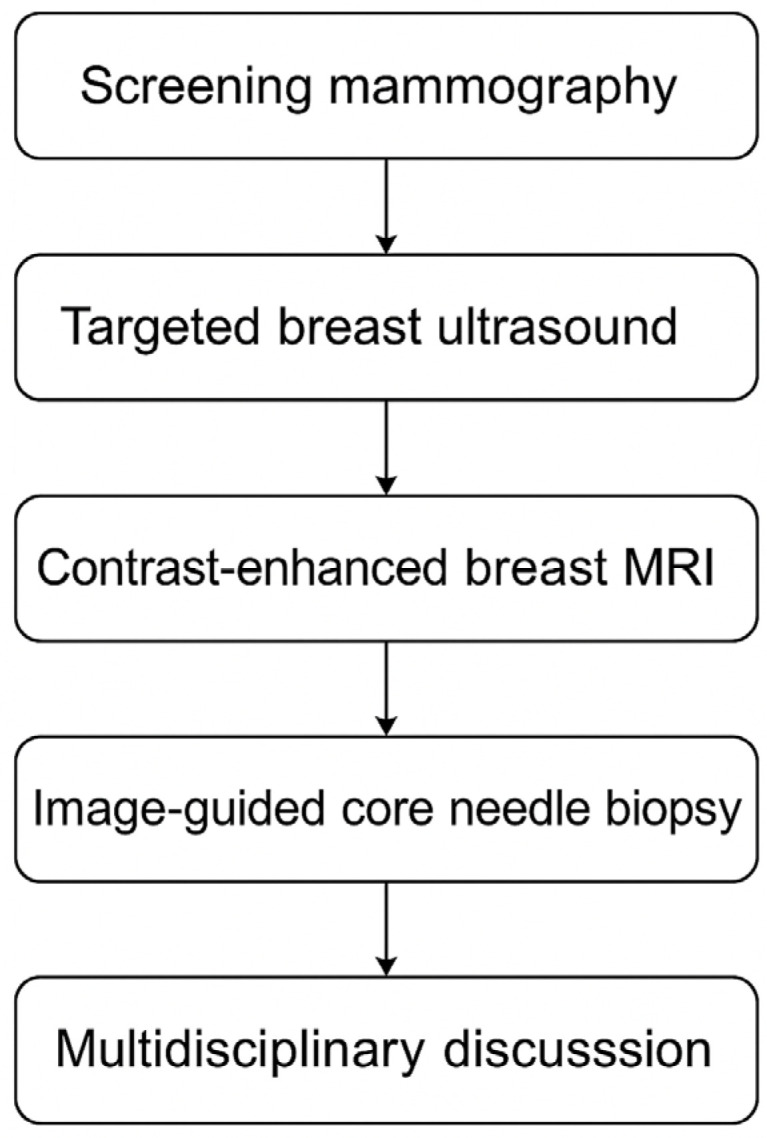
Diagnostic pathway for multifocal and multicentric breast cancer. Sequential imaging evaluation (mammography → ultrasound → MRI) followed by image-guided biopsy ensures accurate detection, staging, and biological characterization of all tumor foci.

When imaging suggests multiple lesions, biopsy is essential. Not all lesions seen on US or MRI are malignant. Image-guided core needle biopsy of suspicious secondary foci is recommended before definitive surgery [1]. In clustered tumors (e.g., satellite nodules), it is common to sample at least one lesion to confirm malignancy. Histological evaluation after surgery remains the gold standard, as some MF cases only become evident on pathology. Studies report that 21.5% of incidental lesions found preoperatively turn out to be malignant. Sentinel lymph node biopsy (SLNB) remains appropriate for MF/MC cases, with accuracy comparable to unifocal cancers. A single peritumoral or periareolar injection is usually sufficient for nodal mapping, as lymphatic drainage generally converges [7]. The diagnostic workflow for multifocal and multicentric breast cancer typically involves a stepwise approach integrating multiple imaging modalities, ensuring accurate lesion detection and characterization (Figure 2).

### 5.3. Imaging Challenges and Limitations

The randomized COMICE trial demonstrated that adding preoperative MRI to conventional imaging did not significantly reduce local recurrence or improve survival, despite increasing mastectomy rates; consequently, current guidelines recommend selective rather than routine preoperative MRI, reserved for cases with lobular histology, dense breasts, or discordant imaging findings. [36] In high-risk populations or uncertain imaging scenarios, MRI’s diagnostic advantages may justify its expense. However, in average-risk patients, the cost-effectiveness of MRI remains limited, as its frequent use can lead to overtreatment without improving long-term outcomes. Therefore, balancing MRI’s diagnostic benefits against financial cost and clinical impact guides its selective application in breast cancer diagnosis and management [1,37,38].

Ultrasound may miss remote lesions and can struggle to differentiate tumor nodules from benign findings like cysts or fibroadenomas. Mammography, though less sensitive, remains crucial for mapping calcifications and identifying multicentric DCIS. Radiologists often integrate mammographic and sonographic findings to determine whether the disease is unifocal or multifocal. In cases with two suspicious, widely separated lesions, biopsy of both and consideration of mastectomy may be justified even before MRI.

In conclusion, optimal diagnosis of MF/MC breast cancer relies on a multimodal imaging approach. Mammography provides a global overview, particularly for calcifications. Ultrasound offers targeted evaluation in dense breasts and around known tumors, and MRI is reserved for selected cases requiring higher sensitivity. Biopsy of additional lesions is critical to avoid overtreatment. Despite advances in imaging, a proportion of multifocal cases are only confirmed by thorough pathological examination of surgical specimens. Collaboration between radiologists, surgeons, and pathologists is vital to accurately assess tumor extent and tailor treatment accordingly.

### 5.4. Advanced Diagnostic and Treatment Strategies of MF/MC Breast Cancer

Implementation of advanced diagnostic and treatment strategies for multifocal and multicentric breast cancer faces several practical challenges. MRI-based screening, despite its improved sensitivity, raises concerns regarding cost-effectiveness, particularly outside high-risk populations such as BRCA mutation carriers or women with very dense breast tissue [39,40]. Financial and resource constraints limit widespread MRI adoption in many healthcare systems, necessitating careful prioritization and risk stratification to optimize benefit. Multidisciplinary coordination, essential for optimal patient management, is often impeded by infrastructural deficits, fragmented communication, and inconsistent access to specialized imaging and molecular diagnostics [41,42,43]. Emerging imaging modalities—such as contrast-enhanced mammography and molecular breast imaging—offer promising avenues for enhanced tumor detection but require rigorous evaluation of clinical impact, logistical feasibility, and equitable deployment across diverse populations [44,45]. Moreover, artificial intelligence (AI) and genomic profiling represent rapidly evolving tools capable of refining risk stratification and therapeutic tailoring for MF/MC tumors, yet challenges remain regarding standardization, validation, and integration into clinical workflows [46]. Addressing these gaps through prospective clinical trials and real-world implementation research will be pivotal in advancing personalized care paradigms and optimizing health outcomes in multifocal and multicentric breast cancer.

## 6. Clinical Management and Treatment

The management of multifocal/multicentric breast cancer follows the same fundamental principles as for unifocal breast cancer—aiming to achieve locoregional control of disease in the breast and regional nodes, and eradication of any micrometastatic disease with systemic therapy. However, the presence of multiple tumor foci can influence the choice of local therapy (surgery and radiation) and may have implications for systemic treatment decisions.

In this narrative review, **adjuvant therapy** is defined as systemic and/or radiotherapeutic treatment administered **postoperatively** with curative intent to eradicate microscopic residual disease. In contrast, **multimodal management** denotes the comprehensive, stage-adapted combination of surgery, systemic therapy (administered in either the neoadjuvant or adjuvant setting), and radiotherapy, coordinated through a multidisciplinary approach. Consistent use of these definitions throughout the manuscript ensures clarity regarding treatment sequencing and therapeutic intent.

### 6.1. Surgical Management of Multifocal and Multicentric Breast Cancer

**Figure 3:**
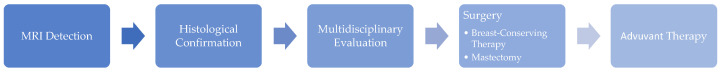
Schematic representation of the diagnostic and therapeutic decision pathway for multifocal and multicentric breast cancer. The flow diagram illustrates the sequence from initial detection through imaging (mammography, ultrasound, or MRI), histologic confirmation by biopsy, multidisciplinary evaluation, surgical decision-making (breast-conserving therapy vs. mastectomy), and subsequent adjuvant management (radiotherapy and/or systemic therapy), followed by structured patient follow-up.

The surgical management of multifocal and multicentric (MF/MC) breast cancer has undergone a fundamental transformation in recent decades. What was once considered an absolute indication for mastectomy has evolved into a nuanced, individualized approach that increasingly embraces breast-conserving strategies when oncological appropriate (Figure 3).

#### 6.1.1. Historical Perspective and Current Practice Patterns

For many years, the presence of multiple tumor foci within the breast automatically relegated patients to mastectomy, based on the presumption that achieving complete excision through breast-conserving surgery would be technically challenging and would inherently carry higher recurrence risks. This conservative surgical philosophy is reflected in contemporary practice patterns, where population-based studies continue to demonstrate that patients with MF/MC disease are substantially more likely to undergo mastectomy compared to their unifocal counterparts. Clinical series have reported mastectomy rates as high as 81% for MF/MC cases, in stark contrast to approximately 45% for unifocal disease, with similar patterns observed across diverse patient populations globally [1].

#### 6.1.2. The Paradigm Shift Toward Breast Conservation

The landscape of MF/MC breast cancer surgery has been revolutionized by accumulating evidence demonstrating that breast-conserving therapy can achieve equivalent oncologic outcomes when all tumor foci can be completely excised with clear margins while maintaining acceptable cosmetic results. This evolution has been facilitated by two crucial developments: the refinement of oncoplastic surgical techniques and the optimization of adjuvant therapeutic protocols [7].

#### 6.1.3. Multifocal Cancers

For multifocal cancers confined within a single quadrant, surgeons have increasingly adopted wide local excision approaches that encompass the entire affected region, essentially performing segmental or quadrant resections. When such procedures achieve complete excision with negative margins, clinical outcomes closely approximate those observed with unifocal lumpectomy. However, the surgical challenge intensifies when the required resection volume becomes disproportionately large relative to breast size, potentially compromising aesthetic outcomes [14]. The integration of oncoplastic surgical principles has dramatically expanded the feasibility of breast-conserving approaches in MF/MC disease. Volume displacement and replacement techniques enable surgeons to reshape the breast following extensive resections, while therapeutic mammoplasty approaches allow for the removal of substantial tissue volumes—including entire quadrants—while preserving breast contour through sophisticated remodeling of remaining tissue [47]. This evolution has given rise to the concept of “extreme oncoplasty,” where surgeons challenge traditional boundaries by combining extensive resections with immediate reconstruction for patients who would historically have required mastectomy. Contemporary case series have demonstrated remarkable success with these approaches, reporting excellent local control rates and high patient satisfaction scores in carefully selected patients with multifocal, multicentric, or large tumors managed through oncoplastic breast-conserving surgery [48]. Despite these advances, important research gaps remain, particularly concerning the optimization of radiotherapy protocols following extensive oncoplastic resections to balance oncologic safety with cosmetic outcomes. Current trials focusing on accelerated partial breast irradiation or hypofractionated schedules in the context of complex oncoplastic surgery may provide crucial insights into minimizing toxicity while maintaining local control. Including such prospective data not only underscores the dynamic nature of MF/MC breast cancer management but also helps identify opportunities for improving multidisciplinary care through integrated surgical and radiation oncology approaches [11].

#### 6.1.4. Multicentric Cancers

Multicentric cancers, involving separate quadrants, have traditionally been managed exclusively with mastectomy due to the technical challenges of multiple excisions and the associated breast deformity. However, selected multicentric cases are now being successfully managed with conservative approaches when tumor burden is limited and breast volume is adequate. These complex procedures may involve multiple separate lumpectomies combined with reduction mammoplasty techniques to restore breast shape and symmetry. The success of such approaches depends on careful case selection, considering factors including the size and number of tumor foci, their anatomical distribution, breast volume, patient preferences, and genetic risk factors. When oncoplastic breast-conserving surgery is attempted but results in positive margins or unacceptable cosmesis, completion mastectomy remains the definitive fallback option.

#### 6.1.5. Evidence Base Supporting Conservative Management

Current clinical guidelines from leading organizations such as the National Comprehensive Cancer Network (NCCN), European Society for Medical Oncology (ESMO), and the UK’s National Institute for Health and Care Excellence (NICE) have incorporated evolving evidence supporting breast-conserving surgery (BCS) as an oncologically safe and feasible option in multifocal and multicentric (MF/MC) breast cancer, provided complete tumor excision with clear margins and appropriate adjuvant therapies are ensured.

The oncologic safety of breast-conserving surgery (BCS) in MF/MC disease has been substantiated by robust clinical evidence. Multiple retrospective series and meta-analyses have consistently demonstrated no significant differences in local recurrence rates or overall survival between MF/MC patients treated with breast-conserving surgery versus mastectomy, provided appropriate adjuvant therapy is administered [47].

For instance, a 2013 Lynch et al. study of 906 MF/MC cases showed 5-year locoregional control ~99% for MF and 96% for MC, statistically similar to unifocal cases, and no independent impact of multifocality on outcomes when treatment type was accounted for [47]. They concluded that BCT is a safe option for MF disease and that multifocality alone is not an indication for post-mastectomy radiation if mastectomy is performed [47]. Likewise, a meta-analysis by Fang et al. (2019) found that while MF/MC patients undergoing BCT had a slightly higher raw local recurrence rate than unifocal BCT (5.6% vs. 4.2%), their outcomes were equivalent to those undergoing mastectomy (no significant difference in recurrence between BCT and mastectomy groups) [10]. ESMO guidelines (2024) emphasize the suitability of BCS in well-selected MF/MC cases, highlighting the importance of multidisciplinary planning and individualized treatment strategies, especially considering tumor volume and patient factors. Similarly, NCCN guidelines (version 3.2024) acknowledge that multifocality alone should not dictate mastectomy, advocating for breast conservation when technically feasible along with whole-breast irradiation and systemic therapy tailored to tumor biology [49,50]. These data have led to contemporary guidelines accepting breast conservation in multifocal/multicentric cancer as long as complete resection is feasible. This could be a major shift from older dogma.

The same optimistic perspective is also shared by Houvenaeghel et al. (2016) when they reviewed the role of breast-conserving surgery (BCS) in multifocal and multicentric breast cancer and concluded that it can be feasible in selected patients [51]. Favorable candidates included women aged 50–69, with limited tumor volume and no extensive DCIS. While MF/MC cancers are associated with higher nodal involvement, local recurrence rates after BCS were low when clear margins and radiotherapy were achieved. The authors emphasized that multidisciplinary planning is essential and that MF/MC status alone should not exclude patients from breast conservation [51].

But recent evidence reinforces the clinical significance of tumor focality in breast cancer prognosis. A comprehensive systematic review and meta-analysis by Zhang et al. (2022) evaluated outcomes in multicentric/multifocal breast cancer (MC/MF-BC) versus unifocal disease [11]. The meta-analysis included 26 studies encompassing 240,146 patients, with 15.5% presenting MC/MF-BC. Results demonstrated a consistently poorer prognosis for MC/MF cases, with significantly increased risks of locoregional recurrence (RR = 1.51, 95% CI: 1.28–1.78) and reduced disease-free survival (HR = 1.38, 95% CI: 1.22–1.56). Overall survival was likewise adversely affected (HR = 1.30, 95% CI: 1.16–1.46). These findings held across various treatment strategies and study designs. Importantly, the analysis underscores the prognostic relevance of multifocality/multicentricity, suggesting that tumor distribution may influence both surgical decision-making and adjuvant therapy planning. Incorporating focality into standard staging and risk assessment algorithms may enhance individualized treatment approaches and improve long-term outcomes [11].

Table 2 summarizes the main differences between breast-conserving therapy and mastectomy in multifocal/multicentric breast cancer, highlighting historical perspectives, current indications, oncological safety, recurrence rates, radiotherapy requirements, cosmetic and psychological outcomes, as well as their position in contemporary guidelines.

#### 6.1.6. Contemporary Indications for Mastectomy

Despite the expanding role of breast conservation, mastectomy remains appropriate and often necessary in specific clinical scenarios. Clear indications include tumors that are too anatomically dispersed to permit excision through a single incision, diffuse malignant-appearing calcifications throughout the breast suggesting extensive multicentric ductal carcinoma in situ, inability to achieve negative margins through breast-conserving approaches, patient preference for more extensive surgery, and genetic mutation carriers who elect for risk-reducing procedures. Additionally, when more than two or three tumor foci are scattered throughout the breast, mastectomy often represents the most prudent surgical course. In such cases, skin-sparing or nipple-sparing mastectomy (NSM) with immediate reconstruction can achieve excellent aesthetic outcomes that rival those of oncoplastic lumpectomy, particularly when the tumor distribution permits preservation of the nipple-areolar complex [52]. Robotic NSM via small axillary incisions has shown feasibility and safety in early reports, enabling NSM plus immediate implant reconstruction in a single session with minimal visible scarring [53].

In summary, recent guideline updates advocate a patient-centered approach to MF/MC breast cancer surgery, favoring breast conservation with appropriate adjuvant therapy when feasible, while reserving mastectomy for specific clinical scenarios. Multidisciplinary collaboration and emerging surgical innovations continue to expand treatment options and improve the quality of life for patients with complex tumor presentations.

#### 6.1.7. Individualized Surgical Decision-Making

The contemporary approach to MF/MC breast cancer surgery emphasizes individualized decision-making that balances oncologic imperatives with patient preferences and aesthetic considerations. Breast-conserving surgery has become increasingly feasible through the systematic application of oncoplastic principles, requiring complete excision of all known tumors with clear margins, typically accomplished through en bloc or segmental resection combined with immediate reconstruction to optimize cosmetic outcomes [54].

The fundamental oncologic principle—complete tumor resection—remains paramount in all surgical planning. When this objective can be achieved through breast-conserving surgery while maintaining acceptable cosmetic results, the presence of multifocality alone no longer constitutes a contraindication to conservative management [6]. Matthijs et al. argue that, when optimal cytoreductive surgery achieves negative margins—including clearance of associated ductal carcinoma in situ (DCIS)—and is integrated with an appropriate imaging strategy (including image-guided localization of nonpalpable lesions), followed by whole-breast irradiation and systemic therapy tailored to tumor biology, local control is favorable, with in-breast recurrence rates <10% at 10 years. Importantly, additional foci detected on magnetic resonance imaging (MRI) without histopathologic confirmation should not, in themselves, prompt mastectomy; MRI findings alone are insufficient to mandate escalation of surgery in the absence of biopsy-proven malignancy [55].

As contemporary surgical philosophy emphasizes, when technically feasible, breast conservation represents an oncologically safe option in MF/MC breast cancer.

### 6.2. Radiation Therapy for MF/MC Breast Cancer

Radiotherapy protocols for multifocal/multicentric (MF/MC) breast cancer adhere to the same principles as unifocal disease, with specific modifications. Following breast-conserving surgery (BCS), whole-breast irradiation (WBI) remains mandatory, targeting all breast tissue harboring potential microscopic disease, which may be more extensive in multifocal presentations. WBI delivers 45 Gy over approximately 5 weeks using conventional fractionation [56], or 3–4 week hypofractionated regimens (WBI/chest wall—26 Gy/5 fractions over 1 week), encompassing the entire breast volume [57].

This is followed by a tumor bed boost dose. A tumor-bed boost reduces in-breast recurrence, largest absolute benefit in younger patients, but increases fibrosis. Classical dosing is 16 Gy/8 fractions after WBI, with patient, tumor, and margin-based selection. The primary consideration in multifocal cases involves boost field selection when multiple tumor foci are resected from different quadrants. Clinical practice typically favors boosting the dominant or largest tumor site. When two distinct lumpectomy cavities are spatially separated, dual-site boosting may be considered, requiring sophisticated treatment planning to minimize toxicity while maintaining dosimetric coverage [58]. Modern radiotherapy techniques enable precise targeting of multiple cavities when clinically indicated. Fortunately, many multifocal cases present with foci within the same quadrant, resulting in a single surgical cavity that requires only one boost field, simplifying treatment delivery and reducing complexity. When multiple lumpectomy sites are present, dual-boost techniques using Intensity-Modulated Radiation Therapy (IMRT) or Volumetric-Modulated Arc Therapy (VMAT) can optimize conformity and minimize toxicity. Partial-breast irradiation remains contraindicated in multifocal or multicentric presentations [59].

#### Post-Mastectomy Radiotherapy in Multifocal/Multicentric Breast Cancer

Post-mastectomy radiotherapy (PMRT) indications for multifocal/multicentric (MF/MC) disease are primarily determined by conventional criteria—tumor size, nodal status, and margin status—rather than multifocality itself. While some clinicians historically considered multicentric disease an independent PMRT indication, current evidence does not support this approach.

Current practice follows standard guidelines: node-positive patients or those with large aggregate tumor burden (≥5 cm) receive PMRT regardless of multifocality. Field design often includes internal mammary and supraclavicular-axillary apical nodes; hypofractionated chest-wall regimens (e.g., 40 Gy/15 or 26 Gy/5 when suitable) are acceptable. Node-negative patients with small tumors do not routinely require PMRT solely based on multifocal presentation, though some oncologists consider multifocality when making borderline decisions [59].

Technical considerations include potential increased toxicity from multiple boost fields, manageable through advanced techniques like intensity-modulated radiotherapy. Partial-breast irradiation remains contraindicated for multifocal disease, necessitating whole-breast treatment to address potential microscopic foci throughout the breast parenchyma [57].

### 6.3. Systemic Therapy (Chemotherapy, Endocrine, Targeted)

Decisions regarding chemotherapy and endocrine therapy in multifocal/multicentric breast cancer generally follow the same principles as for unifocal cancers, being driven by tumor biology (ER/PR status, HER2 status, grade) and stage (particularly lymph node involvement). The presence of multiple foci can influence stage—for staging purposes, the current AJCC/TNM system uses the size of the largest focus as the “T” classification, with a “(m)” modifier to indicate multifocality. All tumor foci in one breast are still considered one cancer when staging; additional foci do not upstage the T category, except insofar as the largest is bigger [60]. The same assertion is further supported by the recent study by Dal et al. [61]. The study included 769 patients (128 MF/MC and 641 UBC) treated surgically from 2006 to 2015. It compared staging based on the largest lesion diameter (“*T-max* stage”) and combined lesion measurements (sum of diameters, area, volume) on disease-free and overall survival. Multivariate analysis identified lymphovascular invasion, estrogen receptor status, nodal involvement, and the “*T-max* stage” as independent prognostic factors for overall survival. The authors concluded that the largest lesion diameter should continue to be used for staging and prognostic classification in MF/MC and unifocal breast cancers [61].

Because of this, systemic therapy recommendations are usually based on the characteristics of the largest (or highest-risk) tumor focus. If any one of the foci has indications for chemotherapy (e.g., triple-negative histology, high grade, large size, or positive nodes), chemotherapy is generally recommended [13]. In essence, the most aggressive focus dictates systemic treatment. This approach is supported by studies on heterogeneity: if at least one focus is HER2-positive, anti-HER2 therapy (e.g., trastuzumab) is indicated, because that HER2+ clone poses a metastatic risk. Similarly, if one tumor is ER-positive and another is ER-negative, endocrine therapy is often still given (if the ER+ tumor is substantial and clinically relevant), but chemotherapy would also be given because of the ER-negative component [23]. In cases where all foci are ER-positive/HER2-negative and node-negative, the indication for adjuvant chemotherapy might be determined by genomic assays (Oncotype DX, etc.) on the highest grade or largest tumor. Interestingly, it has been observed that multifocal tumors tend to have more lymph node positivity; thus, many MF/MC cases are stage II–III and end up receiving chemotherapy for that reason alone [3]. A retrospective analysis by Houvenaeghel et al. found that when MF/MC patients were treated with guideline-adherent adjuvant therapy (chemo, endocrine, etc., as indicated), their survival outcomes could approach those of unifocal patients. If anything, MF/MC patients derive similar or greater benefit from systemic therapy because of their higher baseline risk [11].

For HER2-positive multifocal cancers, anti-HER2 targeted therapy (trastuzumab ± pertuzumab) should be administered, as it would in unifocal HER2+ cancer. The presence of multiple lesions does not change the criteria except ensuring that if any lesion is HER2+ (by either IHC3+ or FISH amplified), the patient is managed as HER2-positive overall [23].

Similarly, endocrine therapy (tamoxifen or aromatase inhibitor) is indicated if the cancer is hormone receptor-positive—in MF/MC, that usually means if the dominant tumor (or any substantial tumor) is ER-positive. It would be unusual to have two sizable tumors where one is ER+ and one ER−, but if that happens, the patient might end up receiving both chemo (for the ER− portion) and endocrine (for the ER+ portion).

One potential consideration in multifocal disease is the use of neoadjuvant therapy. In cases of locally advanced breast cancer, neoadjuvant chemotherapy (NACT) is given to downstage before surgery. Multifocal cancers can certainly be treated with NACT—for example, multiple tumors in one breast that sum to a large volume might prompt chemo first to try to shrink them and enable BCS. However, if tumors are in different quadrants, surgery is likely to be a mastectomy regardless, so neoadjuvant therapy in that case would be aimed more at biologic purposes (e.g., see if HER2+ lesions respond, etc.) rather than breast conservation. In practice, many multifocal tumors are still early-stage (small foci), so upfront surgery is performed, and then adjuvant therapy is decided based on final pathology. If tumors are large or nodes are positive, neoadjuvant systemic therapy is a reasonable approach to potentially reduce tumor extent (possibly converting a needed mastectomy into a lumpectomy). There is nothing contraindicating NACT in MF/MC—they respond just as unifocal tumors do [54].

### 6.4. Other Therapies

In hormonally driven multifocal cancers, ovarian suppression in premenopausal women and other endocrine strategies are applied as usual [62]. In HER2-positive multifocal cancers, newer agents like neratinib (extended adjuvant) or pertuzumab (neoadjuvant/adjuvant) might be used if high-risk (e.g., multiple positive nodes) [63]. If one focus is triple-negative and another is luminal, typically the triple-negative component leads to offering chemotherapy; likewise, immunotherapy (pembrolizumab) in early triple-negative disease is indicated when KEYNOTE-522 criteria are met (e.g., stage II–III, often >2 cm or node-positive) [64]. Essentially, the patient is treated as having one cancer as aggressive as the worst focus, in line with biomarker-driven management and testing standards [65]. It is also worth noting that multifocality is not an explicit variable in widely used prognostic tools like Adjuvant! Online or PREDICT, which primarily incorporate age, tumor size, nodal status, grade, ER/HER2, and treatments [66]. Some researchers have asked whether MF/MC should influence adjuvant chemotherapy for otherwise small, node-negative cancers; for example, two 1 cm ER+ tumors vs. one 2 cm ER+ tumor—current AJCC 8th staging uses the largest focus and the “(m)” suffix, so these are often considered equivalent regarding T category [67]. Ongoing studies of tumor biology and inter-lesional genomic heterogeneity may clarify whether MF/MC warrants distinct systemic strategies [29].

In hormone receptor-positive, HER2-negative early breast cancer, CDK4/6 inhibitors such as abemaciclib have become integral components of adjuvant therapy for patients with high-risk clinicopathologic features, including lymph node involvement, large primary tumors, or high proliferative index. These agents act by selectively inhibiting cyclin-dependent kinases 4 and 6, thereby halting cell-cycle progression and enhancing endocrine responsiveness. The phase III monarchE trial demonstrated that the addition of abemaciclib to standard endocrine therapy significantly improved invasive disease-free survival (IDFS) compared with endocrine therapy alone, with sustained benefit at long-term follow-up [68]. As a result, major international guidelines (ASCO, ESMO, NCCN) now recommend adjuvant CDK4/6 inhibition for eligible patients with high-risk HR+/HER2-disease.

In summary, systemic therapy in multifocal/multicentric breast cancer is based on the most aggressive tumor focus, not multifocality itself. Treatment follows standard criteria (size, nodal status, receptors, grade). All tumor foci must be assessed to guide therapy. High-risk MF/MC patients usually receive adjuvant chemotherapy, with treatments covering all malignant areas, such as HER2-targeted therapy if needed.

## 7. Prognosis

Multiple tumor foci often correlate with greater tumor load and nodal involvement, which are established negative prognostic factors [3]. Neri et al. reported that breast cancer-specific survival (BCSS) at ~12.8 years was significantly shorter for MF/MC patients (median 154 months) than for unicentric cancer patients (204 months) in their series. In their multivariate analysis, the presence of MF/MC tumors remained an independent prognostic factor for worse BCSS, along with positive lymph nodes, ER-negativity, and high Ki-67 [1]. Similarly, some older studies and population datasets have shown higher rates of locoregional recurrence and worse disease-free survival (DFS) in multifocal cases. For instance, Yerushalmi et al. 2012 found that multicentricity was associated with more locoregional recurrence, though not significantly with overall survival, when controlling for tumor size/nodes [28].

On the other hand, a number of studies have failed to find an independent survival disadvantage. The MD Anderson study (Lynch 2013) concluded that MF or MC disease did not independently predict locoregional recurrence or survival after adjusting for tumor size and nodal status [47]. Their 5-year outcomes were virtually identical across MF, MC, and unifocal groups [47]. More recently, a comprehensive systematic review and meta-analysis by Zhang et al. (2022), including 15,703 patients, found no significant difference in overall survival or disease-free survival between multifocal/multicentric and unifocal breast cancers when analyses were adjusted (HR for OS ~1.04, 95% CI 0.96–1.12, not significant) [11]. This suggests that if treated appropriately, patients with multiple foci can do as well as those with one tumor of similar extent. In Zhang’s meta-analysis, MF/MC was associated with poorer prognostic factors (like more nodes, more T3), but it was not itself causing worse outcomes once those factors were accounted for [11].

### 7.1. Local Control Perspective

Earlier studies raised concern that MF/MC disease could lead to higher local recurrence in the breast, especially if treated with BCT. In the modern era, with appropriate radiotherapy, the differences in local recurrence have attenuated. The 2019 systematic review by Fang found the cumulative incidence of local recurrence with BCT was higher in MF/MC (5.6%) than in unifocal (4.2%) at a median follow-up, which was a statistically significant difference. But notably, when they directly compared BCT vs. mastectomy in MF/MC patients, there was no significant difference (i.e., performing BCT did not produce substantially more recurrences than performing a mastectomy for MF/MC cases). This implies that while multifocality might inherently carry a slightly higher risk of residual foci leading to recurrence, effective radiotherapy can mitigate much of that risk, making conservation viable [10].

A comparative summary of the two largest meta-analyses evaluating outcomes in multifocal and multicentric breast cancer is presented in Table 3, highlighting differences in sample size, methodology, and effect estimates for local recurrence and survival.

Moreover, more recent data (Sun et al. 2025 meta-analysis) indicate that the disparity in local recurrence rates between MF/MC and unifocal cases has been diminishing in recent years, likely due to advances in systemic therapy and imaging leading to better margin clearance [69]. Sun et al. reported an odds ratio of 1.76 for local recurrence with MF/MC vs. unifocal BCT (meaning MF/MC had ~1.8 times the odds of recurrence), but importantly, no significant difference in local recurrence between MF/MC patients who had BCT versus those who had mastectomy (OR ~1.72, *p* = 0.07) [69]. Their estimated 5-year DFS and overall survival for MF/MC patients treated with BCT were 88.3% and 95.8%, respectively, which are quite favorable [69].

### 7.2. Survival and Recurrence Rates

Taking a broad view, patients with multifocal/multicentric breast cancer tend to present at a bit higher stage on average—thus, cohorts of MF/MC patients often have lower survival in unadjusted analyses. For example, in Neri’s study, the 10-year BCSS was ~70% for MF/MC vs. ~85% for unifocal (*p* < 0.001), and relapse (any site) was more frequent in MF/MC. However, after adjusting for nodal status and other factors, MF/MC remained prognostic, but it was one factor among many [1]. In other studies (e.g., Wolters 2013 BCRT from Germany’s BRENDA database), MF/MC was associated with more aggressive features but was not an independent predictor of worse survival when treatments were according to guidelines [25]. Zhang’s 2022 meta-analysis even suggested that when appropriately treated, the long-term survival (overall, disease-free, breast cancer-specific) of MF/MC patients is essentially equivalent to unifocal, with hazard ratios close to 1 [11]. This aligns with the view that the key drivers of prognosis are tumor biology and burden (size/nodes), not the focality per se.

Prognostic determinants in multifocal/multicentric (MF/MC) breast cancer broadly parallel those in unifocal disease. Axillary nodal involvement and tumor biology (ER/PR/HER2 status, grade) are the dominant drivers of outcome. The sheer number of foci or the inter-focal distance appears to add limited independent prognostic information; series suggest that having >2 foci correlates with a higher likelihood of residual disease and possibly greater local-recurrence risk, but data remain sparse. By current AJCC/TNM conventions, the T category is assigned by the largest invasive focus with an “(m)” modifier, which preserves prognostic discrimination because the largest diameter correlates with stage [70].

Margin status is crucial in MF breast-conserving surgery: failure to achieve clear margins for all foci increases local-recurrence risk and appropriately triggers re-excision or mastectomy. Composite scores such as the Nottingham Prognostic Index tend to be higher in MF/MC because they incorporate size and nodal status, not multifocality per se. Overall, MF/MC presentation is associated with slightly higher locoregional risk and more adverse pathology, but with complete resection and appropriate adjuvant therapy, outcomes approach those of unifocal diseas. Five-year survival typically exceeds 90% in early-stage multifocal and multicentric breast cancer when guideline-concordant multimodal therapy is applied [7].

### 7.3. Psychosocial Implications of Multiple Tumors

Psychosocial impact and quality-of-life impairment represent key yet often underappreciated dimensions of multifocal and multicentric breast cancer. Patients frequently experience heightened anxiety, depression, and decisional distress due to the complexity of their disease and the frequent indication for mastectomy or extensive systemic therapy [71]. Body image concerns and altered self-perception are particularly pronounced after bilateral or reconstructive surgery, affecting emotional well-being and sexual health [72]. Evidence shows that structured psychological support, including cognitive-behavioral interventions and group-based counseling, significantly improves adjustment and treatment adherence [73].

Psychological support and counseling are essential to enhance quality of life and adherence to long-term treatments [74]. Psychosocial interventions, including cognitive behavioral therapy and mindfulness approaches, have demonstrated improvements in distress and quality of life outcomes in breast cancer cohorts [75]. In long-term survivors, sociodemographic and clinical factors modulate residual quality-of-life deficits, with patients reporting persistent fatigue, sleep disturbance, and emotional distress even years post-treatment [74].

## 8. Controversies and Perspectives

### 8.1. Definitional Debates

A persistent barrier to evidence synthesis is the absence of uniform definitions distinguishing multifocal (MF) from multicentric (MC) disease. Studies variably apply distance thresholds (e.g., 2 cm vs. 5 cm), quadrant-based mapping, or ductal-system criteria, yielding non-comparable cohorts. Standardization proposals include adopting the umbrella term “multiple ipsilateral breast cancers (MIBC)” with subclassification by distance/quadrant [3]. Staging remains contentious: current TNM assigns T by the largest invasive focus with an “(m)” descriptor; suggestions to incorporate number of foci or composite size/volume have not been adopted due to uncertain incremental prognostic value [60,70].

A major controversy concerns the staging of MF/MC breast cancers: whether only the largest tumor focus (*T-max*) should be considered, or whether aggregate tumor size/volume should be included.

Proposals to incorporate aggregate diameter/total tumor volume are intuitively appealing. Indeed, summing diameters upstaged ~14% of MF cases, yet 10-year overall survival remained comparable to their original stage cohorts, implying limited incremental prognostic yield once standard factors are accounted for [60].

Several studies have addressed this issue with divergent conclusions (Table 4).

So, some investigations advocate aggregate diameter measurement better reflects total tumor burden and provides superior prognostic stratification, while others support largest tumor diameter as the most reliable survival predictor. Pathologist surveys reveal substantial interobserver variability with no consensus among specialists. This uncertainty reflects molecular heterogeneity between tumor foci and variable aggressive behavior patterns. Standardized guidelines incorporating pathological and clinical factors remain urgently needed for optimal therapeutic decision-making. In this moment Internationally accepted standard is AJCC/TNM witch is a pragmatic approach, offers the best correlation with prognosis, and avoids overstaging.

### 8.2. Clinical Management Controversies

The historical question—whether BCT is appropriate for MF/MC-persists. While accumulating data support BCT in selected patients (often with oncoplastic techniques), practice patterns still diverge, especially for MC [10]. Post-mastectomy radiotherapy (PMRT) in node-negative MF/MC is another gray area; guidelines do not recommend routine PMRT, yet registry analyses continue to probe whether multifocality independently elevates risk in borderline scenarios. Tumor boards frequently revisit these thresholds [57].

### 8.3. Pathology Considerations

Specimen handling influences the detection of additional foci. Advocates of large-format or more exhaustive sectioning argue that it reduces missed satellites, particularly in partial mastectomy specimens. The classification and clinical significance of multifocal DCIS accompanying invasive disease remain debated, with implications for margin assessment and re-excision strategy [27,28].

### 8.4. Gaps in Research

Key uncertainties include: the molecular basis of multifocality (clonality, drivers such as E-cadherin loss in lobular carcinoma), understudied psychosocial impact and patient-reported outcomes (anxiety, preference for mastectomy despite BCT eligibility), and limited outcomes data in the genomic-assay era (e.g., whether low-risk signatures truly neutralize MF/MC risk). In radiation oncology, unresolved issues include the role, if any, of partial-breast irradiation in tightly clustered MF and optimal boost strategies when two beds require coverage (simultaneous integrated vs. sequential).

### 8.5. Future Research Directions

Priorities include: refining risk-adapted de-escalation (when multifocality does not worsen survival after systemic therapy) versus intensification for heterogeneously aggressive foci or marginal clearance concerns; evaluating emerging imaging (contrast-enhanced mammography, molecular breast imaging) to stage MF/MC with fewer false positives than MRI; establishing long-term oncologic safety of “extreme oncoplasty” compared with mastectomy; testing “field” treatments (e.g., intraoperative or wider-field RT) to sterilize occult multifocality; integrating prevention and genetic risk assessment given potential contralateral risk; and operationalizing intratumoral heterogeneity and liquid biopsy (ctDNA) to detect minimal residual disease and tailor adjuvant therapy.

In conclusion, management has shifted from default mastectomy to individualized, multidisciplinary care. Progress now depends on standardized definitions, better molecular and patient-reported endpoints, and prospective trials that clarify when conservation is safe, how systemic therapy should or should not change for MF/MC, and how to optimize radiation planning in multifocal anatomy.

## 9. Conclusions

Multifocal/multicentric breast cancer signals greater tumor burden and nodal involvement, yet focality itself is not the dominant driver of prognosis. When treatment is stage-matched and guideline-concordant, survival and disease control approximate those of unifocal disease. The determinants that matter most are biology (ER/PR/HER2 status, grade) and extent (tumor size, nodal status). Breast-conserving therapy remains acceptable in carefully selected patients, provided all foci can be removed with negative margins and whole-breast irradiation is delivered; mastectomy does not inherently confer superior local control. Staging by the largest invasive focus with an “m” descriptor retains practical prognostic value; aggregate size or volume can inform judgment but rarely changes outcomes once core factors are addressed. Achieving clear margins across all tumor beds is critical; failure warrants timely re-excision or conversion to mastectomy. Finally, the psychosocial load is substantial; proactive, tailored counseling improves decision quality, treatment adherence, body-image adjustment, and overall quality of life.

This review closes several gaps left by earlier overviews: (i) it operationalizes **ultra-hypofractionated** whole-breast schedules and **boost strategies** in MF/MC planning; (ii) it appraises **extreme oncoplasty** as a limb-sparing alternative to mastectomy in selected MF/MC configurations; and (iii) it incorporates **multi-lesion genomic profiling**, highlighting clinically relevant inter-lesional divergence (e.g., discordant copy-number events) that can upstage systemic therapy. Collectively, these updates **recalibrate** expectations—MF/MC entails slightly higher in-breast event risk, yet **stage- and biology-adjusted survival** approaches that of unifocal disease in modern care pathways.

## Figures and Tables

**Table 1:** Differences in terms of incidence, imaging features, surgical/systemic treatment, histopathological findings, and prognosis in multifocal (MF), multicentric (MC), and unifocal breast cancer.

Entity	Incidence	Mammography/MRI	Surgical Treatment	Systemic Treatment	Histopathologic Findings	Prognosis
Multifocal (MF)	~10–20% overall when modern imaging is used; often underdetected without MRI.	Mammography/US: moderate sensitivity; MRI reveals additional foci in ~10–30%.	Breast-conserving therapy (BCT) is feasible if all foci can be excised with negative margins; oncoplastic approaches reduce cosmetic penalty.	Biology-driven (ER/PR/HER2, grade, nodes). Treat to the highest-risk focus: anti-HER2 for HER2+, chemo for TNBC, endocrine for ER+.	Multiple foci within one quadrant; often clonal origin; occasional receptor discordance between foci.	Slightly higher in-breast recurrence with BCT vs. unifocal (e.g., ~5.6% vs. ~4.2%), yet adjusted OS/DFS comparable to unifocal when guideline-concordant multimodal therapy is delivered.
Multicentric (MC)	~5–10% depending on definition (different quadrants or ≥5 cm apart); rarer than MF.	Conventional imaging underestimates dispersion; MRI and whole-organ pathology markedly increase detection.	Historically, mastectomy; carefully selected cases may undergo oncoplastic BCT with image-guided localization; margin control is challenging.	Same principles as MF; therapy aligned to the most aggressive clone; node positivity rates higher (~up to 45%), prompting more frequent adjuvant chemo.	Lesions in different quadrants; higher probability of independent primaries; receptor heterogeneity is more common.	Historically worse BCSS in some series, but modern adjusted analyses show no independent survival disadvantage vs. unifocal; BCT and mastectomy yield similar local control when radiotherapy is appropriate.
Unifocal	Most common presentation (~70–80% of breast cancers).	Mammography/US usually sufficient; MRI selectively used (e.g., dense breasts, lobular histology, staging discordance).	BCT or mastectomy per standard criteria (size-to-breast ratio, margins, patient preference); lowest re-excision rates versus MF/MC.	Standard stage- and biology-adapted systemic therapy; fewer discordant biomarkers than in MF/MC.	Single lesion; typically, homogeneous receptors; lower nodal positivity (~25–28%).	Favorable local control and survival; outcomes primarily driven by tumor biology and nodal status.

MF—Multifocal; MC—Multicentric; MRI—Magnetic Resonance Imaging; BCS—Breast-Conserving Surgery; SLNB—Sentinel Lymph Node Biopsy; ALND—Axillary Lymph Node Dissection; ER—Estrogen Receptor; PR—Progesterone Receptor; HER2—Human Epidermal Growth Factor Receptor 2; OS—Overall Survival; DFS—Disease-Free Survival.

**Table 2:** Comparative Table—Conservative Surgery (BCT) vs. Mastectomy in MF/MC Breast Cancer.

Aspect	Breast-Conserving Therapy (BCT)	Mastectomy
Historical perspective	Historically contraindicated due to concern of residual disease and higher recurrence	Considered the standard approach for MF/MC for many years
Current indications	Feasible when all foci can be excised with negative margins and acceptable cosmesis; often aided by oncoplastic techniques	Indicated when tumors are widely dispersed, margins cannot be achieved, extensive calcifications, >2–3 foci, genetic risk, or patient preference
Oncological safety	Contemporary studies and meta-analyses show equivalent overall survival and disease-free survival compared to mastectomy, if margins clear and radiotherapy applied	Provides reliable local control; outcomes equivalent to BCT when modern radiotherapy is used
Local recurrence rates	Slightly higher after BCT in MF/MC (≈5.6%) vs. unifocal (≈4.2%); however, not significantly different compared with mastectomy in MF/MC patients	Historically thought to reduce recurrence, but recent evidence shows no superiority over BCT if radiotherapy is delivered
Role of radiotherapy	Whole-breast irradiation mandatory; boost usually to largest or dominant cavity; complex planning if multiple cavities	Post-mastectomy radiotherapy (PMRT) indicated only by classical criteria (tumor size, nodal status, margins), not by multifocality per se
Cosmetic outcomes	Preservation of breast; oncoplastic/extreme oncoplastic techniques improve aesthetics even with larger resections	Requires reconstruction for cosmetic results; nipple/skin-sparing mastectomy with immediate reconstruction can yield good aesthetics
Psychological impact	Better body image and quality of life in many patients	Often associated with greater psychological burden, anxiety, body image disturbance
Guideline position	Increasingly accepted as safe and effective for selected MF/MC cases with adequate surgery and adjuvant therapy	Still preferred in anatomically complex cases, high tumor burden, or by patient choice

BCT = Breast-Conserving Therapy; MF = Multifocal; MC = Multicentric; PMRT = Post-Mastectomy Radiotherapy.

**Table 3:** Comparative summary of major meta-analyses evaluating outcomes in multifocal and multicentric breast cancer.

Study (Year)	Study Type	No. of Patients	Key Outcomes	Effect Size (95% CI)	Main Conclusion
Fang et al., 2019 (Breast Care) [10]	Systematic review & meta-analysis (8 studies)	7297	Local recurrence after BCT vs. mastectomy	RR = 1.33 (95% CI 1.08–1.63), *p* = 0.006	Slightly higher in-breast recurrence with BCT (5.6% vs. 4.2%), but no survival disadvantage when radiotherapy was applied.
Zhang et al., 2022 (Front Oncol) [11]	Systematic review & meta-analysis (26 studies)	240,146	DFS and OS in MF/MC vs. unifocal BC	HR = 1.38 (DFS, 95% CI 1.22–1.56); HR = 1.30 (OS, 95% CI 1.16–1.46)	Slightly poorer DFS/OS in unadjusted analyses; no independent prognostic impact after stage and nodal adjustment.

BCT, breast-conserving therapy; BC, breast cancer; CI, confidence interval; DFS, disease-free survival; HR, hazard ratio; MF/MC, multifocal/multicentric; OS, overall survival; RR, relative risk.

**Table 4:** Comparative Table—Controversial Opinions on Staging in MF/MC ^1^ Breast Cancer.

Author/Study	Opinion on Staging	Main Arguments	Conclusion
AJCC/TNM (current system)	Uses only the largest tumor focus (*T-max*), with the suffix “(m)”	Pragmatic approach; best correlation with prognosis; avoids overstaging	Internationally accepted standard
Dal et al., 2024 [61]	Compared *T-max* with cumulative measures (sum of diameters, area, volume)	*T-max* remained an independent prognostic factor for OS; cumulative metrics added limited prognostic value	Supports *T-max*
Rezo et al., 2011 [24]	Proposed incorporating total aggregate size	MF/MC cases carry higher tumor burden → worse prognosis compared to unifocal cancers with same *T-max*	Argues for revising staging criteria
Boyages et al., 2010 [12]	“Each focus matters”	MF/MC cancers associated with worse survival, particularly in larger tumors	Ignoring additional foci may underestimate risk
Zhang et al., 2022 (meta-analysis) [11]	Adjusted analyses showed MF/MC did not independently affect OS/DFS beyond T and N stage	Prognostic differences disappear when adjusted for tumor size and nodal status	Confirms validity of *T-max* staging
Wolters et al., 2013 (BRENDA cohort) [25]	MF/MC tumors display more aggressive features	Prognosis still primarily determined by T and N stage rather than multiplicity	Endorses *T-max* + “(m)” suffix
Recent biological proposals (e.g., sick lobe hypothesis, field effect—Tan et al., 2023) [32]	Suggest staging should consider total tumor volume and clonal biology	Biological rationale: MF usually reflects intraductal spread; MC may represent independent primaries	No consensus; exploratory research direction

^1^ MF = Multifocal; MC = Multicentric; OS = Overall Survival; DFS = Disease-Free Survival; AJCC = American Joint Committee on Cancer; TNM = Tumor-Node-Metastasis classification.

## References

[1] Neri A., Marrelli D., Megha T., Bettarini F., Tacchini D., De Franco L., Roviello F. (2015). Clinical significance of multifocal and multicentric breast cancers and choice of surgical treatment: A retrospective study on a series of 1158 cases. BMC Surg..

[2] Mohamed Nazir M.H., Ismail M.S., Ismail I.S., Ghazali M.F., Thaumanavar C.E., Rahman K.S.A., Rahman W.I.W.A. (2020). Multicentric breast cancer comprising of three different histopathological types: A case report. Ann. Breast Surg..

[3] Zhou M.R., Tang Z.H., Li J., Fan J.H., Pang Y., Yang H.J., Zheng S., Bai J.Q., Lv N., Qiao Y.L. (2013). Clinical and Pathologic Features of Multifocal and Multicentric Breast Cancer in Chinese Women: A Retrospective Cohort Study. J. Breast Cancer.

[4] Pekar G., Hofmeyer S., Tabár L., Tarján M., Chen T.H., Yen A.M., Chiu S.Y., Hellberg D., Gere M., Tot T. (2013). Multifocal breast cancer documented in large-format histology sections. Cancer.

[5] Jesinger R.A. (2014). Breast Anatomy for the Interventionalist. Tech. Vasc. Interv. Radiol..

[6] Zhu H., Doğan B.E. (2021). American Joint Committee on Cancer’s Staging System for Breast Cancer, Eighth Edition: Summary for Clinicians. Eur. J. Breast Health.

[7] Masannat Y.A., Agrawal A., Maraqa L., Fuller M., Down S.K., Tang S., Pang D., Kontos M., Romics L., Heys S.D. (2020). Multifocal and multicentric breast cancer, is it time to think again?. Ann. R. Coll. Surg. Engl..

[8] Avera E., Valentic L., Bui L. (2023). Current understanding and distinct features of multifocal and multicentric breast cancers. Cancer Rep..

[9] Vera-Badillo F.E., Napoleone M., Ocana A., Templeton A.J., Seruga B., Al-Mubarak M., AlHashem H., Tannock I.F., Amir E. (2014). Effect of multifocality and multicentricity on outcome in early stage breast cancer: A systematic review and meta-analysis. Breast Cancer Res. Treat..

[10] Fang M., Zhang X., Zhang H., Wu K., Yu Y., Sheng Y. (2019). Local Control of Breast Conservation Therapy versus Mastectomy in Multifocal or Multicentric Breast Cancer: A Systematic Review and Meta-Analysis. Breast Care.

[11] Zhang Y., Liu F., Gao Q., Chai Y., Ren Y., Tian H., Ma B., Song A. (2022). Comparing the outcome between multicentric/multifocal breast cancer and unifocal breast cancer: A systematic review and meta-analysis. Front. Oncol..

[12] Boyages J., Jayasinghe U.W., Coombs N. (2010). Multifocal breast cancer and survival: Each focus does matter particularly for larger tumours. Eur. J. Cancer.

[13] Beiranvand M., Akbari A., Akbari M.E. (2025). Evaluation of recurrence and survival in multifocal versus unifocal breast cancer patients at a tertiary center: A case-control study. Cancer Treat. Res. Commun..

[14] Lynch S.P., Lei X., Chavez-MacGregor M., Hsu L., Meric-Bernstam F., Buchholz T.A., Zhang A., Hortobagyi G.N., Valero V., Gonzalez-Angulo A.M. (2012). Multifocality and multicentricity in breast cancer and survival outcomes. Ann. Oncol..

[15] Anderson K.N., Schwab R.B., Martinez M.E. (2014). Reproductive risk factors and breast cancer subtypes: A review of the literature. Breast Cancer Res. Treat.

[16] Aktas A., Gurleyik M.G., Akkus D., Ucur Z., Aker F. (2025). Invasive lobular breast carcinoma variants; clinicopathological features and patient outcomes. Breast Cancer Res. Treat.

[17] Radswiki T., Niknejad M.T., Shaggah M. (2011). Invasive lobular carcinoma of the breast. Radiopaedia.org..

[18] Weinstein S.P., Orel S.G., Heller R., Reynolds C., Czerniecki B., Solin L.J., Schnall M. (2001). MR Imaging of the Breast in Patients with Invasive Lobular Carcinoma. Am. J. Roentgenol..

[19] Kuchenbaecker K.B., Hopper J.L., Barnes D.R., Phillips K.A., Mooij T.M., Roos-Blom M.J., Jervis S., van Leeuwen F.E., Milne R.L., Andrieu N. (2017). Risks of Breast, Ovarian, and Contralateral Breast Cancer for *BRCA1* and *BRCA2* Mutation Carriers. JAMA.

[20] Steinhof-Radwańska K., Lorek A., Holecki M., Barczyk-Gutkowska A., Grażyńska A., Szczudło-Chraścina J., Bożek O., Habas J., Szyluk K., Niemiec P. (2021). Multifocality and Multicentrality in Breast Cancer: Comparison of the Efficiency of Mammography, Contrast-Enhanced Spectral Mammography, and Magnetic Resonance Imaging in a Group of Patients with Primarily Operable Breast Cancer. Curr. Oncol..

[21] Francis A., Thomas J., Fallowfield L., Wallis M., Bartlett J.M., Brookes C., Roberts T., Pirrie S., Gaunt C., Young J. (2015). Addressing overtreatment of screen detected DCIS; the LORIS trial. Eur. J. Cancer.

[22] Bozzini A., Renne G., Meneghetti L., Bandi G., Santos G., Vento A.R., Menna S., Andrighetto S., Viale G., Cassano E. (2008). Sensitivity of imaging for multifocal-multicentric breast carcinoma. BMC Cancer.

[23] Li S., Wu J., Huang O., He J., Chen W., Li Y., Chen X., Shen K. (2022). Association of Molecular Biomarker Heterogeneity with Treatment Pattern and Disease Outcomes in Multifocal or Multicentric Breast Cancer. Front. Oncol..

[24] Rezo A., Dahlstrom J., Shadbolt B., Rodins K., Zhang Y., Davis A.J. (2011). Tumor size and survival in multicentric and multifocal breast cancer. Breast.

[25] Wolters R., Wöckel A., Janni W., Novopashenny I., Ebner F., Kreienberg R., Wischnewsky M., Schwentner L., BRENDA Study Group (2013). Comparing the outcome between multicentric and multifocal breast cancer: What is the impact on survival, and is there a role for guideline-adherent adjuvant therapy? A retrospective multicenter cohort study of 8935 patients. Breast Cancer Res. Treat..

[26] Iorfida M., Maiorano E., Orvieto E., Maisonneuve P., Bottiglieri L., Rotmensz N., Montagna E., Dellapasqua S., Veronesi P., Galimberti V. (2012). Invasive lobular breast cancer: Subtypes and outcome. Breast Cancer Res. Treat..

[27] Pekár G., Gere M., Tarjan M., Hellberg D., Tot T. (2014). Molecular phenotype of the foci in multifocal invasive breast carcinomas: Intertumoral heterogeneity is related to shorter survival and may influence the choice of therapy. Cancer.

[28] Yerushalmi R., Tyldesley S., Woods R., Kennecke H.F., Speers C., Gelmon K.A. (2012). Is breast-conserving therapy a safe option for patients with tumor multicentricity and multifocality?. Ann. Oncol..

[29] Ahn S., Kim H.J., Kang E., Kim E.K., Kim S.H., Kim J.H., Kim I.A., Park S.Y. (2020). Genomic profiling of multiple breast cancer reveals inter-lesional heterogeneity. Br. J. Cancer.

[30] Jeon T., Kim H., Kim A., Kim C. (2022). A systematic study on phenotypical characteristics of invasive breast carcinoma and surrounding ductal carcinoma in situ in multifocal breast cancers. Hum. Pathol..

[31] Pekar G., Davies H., Lukacs A.P., Forsberg L., Hellberg D., Dumanski J., Tot T. (2016). Biobanking multifocal breast carcinomas: Sample adequacy with regard to histology and DNA content. Histopathology.

[32] Tan M.P., Sitoh Y.Y. (2023). The unifying concepts of the sick lobe hypothesis, field cancerisation and breast conservation treatment for multiple ipsilateral breast cancers: A narrative review. Gland. Surg..

[33] Madjar H. (2010). Role of Breast Ultrasound for the Detection and Differentiation of Breast Lesions. Breast Care.

[34] Sardanelli F., Boetes C., Borisch B., Decker T., Federico M., Gilbert F.J., Helbich T., Heywang-Köbrunner S.H., Kaiser W.A., Kerin M.J. (2010). Magnetic resonance imaging of the breast: Recommendations from the EUSOMA working group. Eur. J. Cancer.

[35] Milulescu A., Di Marino L., Peradze N., Toesca A. (2017). Management of Multifocal-Multicentric Breast Cancer: Current Perspective. Chirurgia.

[36] Turnbull L., Brown S., Harvey I., Olivier C., Drewc P., Napp V., Hanby A., Brown J. (2010). Comparative effectiveness of MRI in breast cancer (COMICE) trial: A randomised controlled trial. Lancet.

[37] Tollens F., Baltzer P.A.T., Froelich M.F., Kaiser C.G. (2023). Economic evaluation of breast MRI in screening—A systematic review and basic approach to cost-effectiveness analyses. Front. Oncol..

[38] Ghorbani S., Rezapour A., Eisavi M., Barahman M., Bagheri Faradonbeh S. (2023). Cost-benefit Analysis of Breast Cancer Screening with Digital Mammography: A Systematic Review. Med. J. Islam. Repub. Iran..

[39] Geuzinge H.A., Bakker M.F., Heijnsdijk E.A.M., van Ravesteyn N.T., Veldhuis W.B., Pijnappel R.M., de Lange S.V., Emaus M.J., Mann R.M., Monninkhof E.M. (2021). Cost-Effectiveness of Magnetic Resonance Imaging Screening for Women with Extremely Dense Breast Tissue. J. Natl. Cancer Inst..

[40] Hill H., Roadevin C., Duffy S., Mandrik O., Brentnall A. (2024). Cost-Effectiveness of AI for Risk-Stratified Breast Cancer Screening. JAMA Netw. Open..

[41] European Oncology Nursing Society POLICY STATEMENT ON MULTIDISCIPLINARY CANCER CARE. https://www.oeci.eu/Attachments%5CPolicy_Statement__Multidisciplinary_Cancer_Care_02-12.pdf.

[42] Kočo L., Weekenstroo H.H.A., Lambregts D.M.J., Sedelaar J.P.M., Prokop M., Fütterer J.J., Mann R.M. (2021). The Effects of Multidisciplinary Team Meetings on Clinical Practice for Colorectal, Lung, Prostate and Breast Cancer: A Systematic Review. Cancers.

[43] UK National Screening Committee (2025). Risk-Adapted Breast Imaging in Population Breast Cancer Screening: A UK National Screening Committee Evidence Summary. Version 1.

[44] National Breast Screening Program (2023). National policies for screening and early detection of breast cancer. Int. J. Gynaecol. Obstet..

[45] Zhang H., Yang F., Xu Y., Zhao S., Jiang Y.Z., Shao Z.M., Xiao Y. (2025). Multimodal integration using a machine learning approach facilitates risk stratification in HR^+^/HER^2−^ breast cancer. Cell Rep. Med..

[46] Goyal M., Marotti J.D., Workman A.A., Tooker G.M., Ramin S.K., Kuhn E.P., Chamberlin M.D., diFlorio-Alexander R.M., Hassanpour S. (2024). A multi-model approach integrating whole-slide imaging and clinicopathologic features to predict breast cancer recurrence risk. NPJ Breast Cancer.

[47] Lynch S.P., Lei X., Hsu L., Meric-Bernstam F., Buchholz T.A., Zhang H., Hortobágyi G.N., Gonzalez-Angulo A.M., Valero V. (2013). Breast Cancer Multifocality and Multicentricity and Locoregional Recurrence. Oncologist.

[48] Savioli F., Seth S., Morrow E., Doughty J., Stallard S., Malyon A., Romics L. (2021). Extreme Oncoplasty: Breast Conservation in Patients with Large, Multifocal, and Multicentric Breast Cancer. Breast Cancer Targets Ther..

[49] European Society for Medical Oncology (ESMO) (2024). Breast Cancer: ESMO Clinical Practice Guidelines. https://www.esmo.org/guidelines.

[50] National Comprehensive Cancer Network NCCN Clinical Practice Guidelines in Oncology: Breast Cancer, Version 3.2024. https://www.nccn.org/professionals/physician_gls/pdf/breast.pdf.

[51] Houvenaeghel G., Tallet A., Jalaguier-Coudray A., Cohen M., Bannier M., Jauffret-Fara C., Lambaudie E. (2016). Is breast conservative surgery a reasonable option in multifocal or multicentric tumors?. World J. Clin. Oncol..

[52] Botteri E., Rotmensz N., Sangalli C., Toesca A., Peradze N., De Oliveira Filho H.R., Sagona A., Intra M., Veronesi P., Galimberti V. (2009). Unavoidable mastectomy for ipsilateral breast tumour recurrence after conservative surgery: Patient outcome. Ann. Oncol..

[53] Toesca A., Peradze N., Manconi A., Galimberti V., Intra M., Colleoni M., Bonanni B., Curigliano G., Rietjens M., Viale G. (2017). Robotic nipple-sparing mastectomy for the treatment of breast cancer: Feasibility and safety study. Breast.

[54] Koppiker C.B., Noor A.U., Dixit S., Busheri L., Sharan G., Dhar U., Allampati H.K., Nare S. (2019). Extreme Oncoplastic Surgery for Multifocal/Multicentric and Locally Advanced Breast Cancer. Int. J. Breast Cancer.

[55] Nijenhuis M.V., Emiel J. (2015). Conservative surgery for multifocal/multicentric breast cancer. Breast.

[56] Smith B.D., Bellon J.R., Blitzblau R., Freedman G., Haffty B., Hahn C., Halberg F., Hoffman K., Horst K., Moran J. (2018). Radiation therapy for the whole breast: Executive summary of an American Society for Radiation Oncology (ASTRO) evidence-based guideline. Pract. Radiat. Oncol..

[57] Murray Brunt A., Haviland J.S., Wheatley D.A., Sydenham M.A., Alhasso A., Bloomfield D.J., Chan C., Churn M., Cleator S., Coles C.E. (2020). Hypofractionated breast radiotherapy for 1 week versus 3 weeks (FAST-Forward): 5-year efficacy and late normal tissue effects results from a multicentre, non-inferiority, randomised, phase 3 trial. Lancet.

[58] Bartelink H., Maingon P., Poortmans P., Weltens C., Fourquet A., Jager J., Schinagl D., Oei B., Rodenhuis C., Horiot J.C. (2015). Whole-breast irradiation with or without a boost for patients treated with breast-conserving surgery for early breast cancer: 20-year follow-up of a randomised phase 3 trial. Lancet Oncol..

[59] Recht A., Comen E.A., Fine R.E., Fleming G.F., Hardenbergh P.H., Ho A.Y., Hudis C.A., Hwang E.S., Kirshner J.J., Morrow M. (2016). Postmastectomy Radiotherapy: An American Society of Clinical Oncology, American Society for Radiation Oncology, and Society of Surgical Oncology Focused Guideline Update. Pract. Radiat. Oncol..

[60] Karakas Y., Dizdar O., Akin S., Ates O., Sendur M.A.N., Aksoy S., Hayran M., Gullu I.H., Ozisik Y.Y., Altundag K. (2016). The impact of the total size of lesions in multifocal/multicentric breast cancer on survival. J. Clin. Oncol..

[61] Dal F., Ökmen H., Ulusan K., Battal Havare S., Sari S. (2024). The effect of total size, area, and volume of lesions in multifocal/multicentric breast cancers and unifocal breast cancers on survival: An observational study. Medicine.

[62] Francis P.A., Pagani O., Fleming G.F., Walley B.A., Colleoni M., Láng I., Gómez H.L., Tondini C., Ciruelos E., Burstein H.J. (2018). Tailoring Adjuvant Endocrine Therapy for Premenopausal Breast Cancer. N. Engl. J. Med..

[63] Piccart M., Procter M., Fumagalli D., de Azambuja E., Clark E., Ewer M.S., Restuccia E., Jerusalem G., Dent S., Reaby L. (2021). Adjuvant Pertuzumab and Trastuzumab in Early HER2-Positive Breast Cancer in the APHINITY Trial: 6 Years’ Follow-Up. J. Clin. Oncol..

[64] Schmid P., Cortes J., Dent R., McArthur H., Pusztai L., Kümmel S., Denkert C., Park Y.H., Hui R., Harbeck N. (2024). Overall Survival with Pembrolizumab in Early-Stage Triple-Negative Breast Cancer. N. Engl. J. Med..

[65] Allison K.H., Hammond M.E.H., Dowsett M., McKernin S.E., Carey L.A., Fitzgibbons P.L., Hayes D.F., Lakhani S.R., Chavez-MacGregor M., Perlmutter J. (2020). Estrogen and Progesterone Receptor Testing in Breast Cancer: ASCO/CAP Guideline Update. J. Clin. Oncol..

[66] Ravdin P.M., Siminoff L.A., Davis G.J., Mercer M.B., Hewlett J., Gerson N., Parker H.L. (2001). Computer Program to Assist in Making Decisions About Adjuvant Therapy for Women with Early Breast Cancer. J. Clin. Oncol..

[67] Tong Y., Sun F., Zhang C., Yang S., Yu Z., Zhao Y. (2023). Multifocal/multicentric breast cancer: Does each focus matter?. Cancer Med..

[68] Johnston S.R.D., Toi M., O’Shaughnessy J., Rastogi P., Campone M., Neven P., Huang C.S., Huober J., Jaliffe G.G., Cicin I. (2023). Abemaciclib plus endocrine therapy for hormone receptor-positive, HER2-negative, node-positive, high-risk early breast cancer (monarchE): Results from a preplanned interim analysis of a randomised, open-label, phase 3 trial. Lancet Oncol..

[69] Sun Y., Gao L., Zhou X., Wang Z., Li Y., Sun Q. (2025). Local Recurrence and Survival Outcomes of Multifocal/Multicentric Breast Cancer After Breast Conserving Therapy: A systematic Review and Meta-Analysis. Clin. Breast Cancer.

[70] Boros M., Moldovan C., Varlam C.M., Podoleanu C., Georgescu R., Stolnicu S. (2016). Which is the best method to measure the size in multiple breast carcinoma in correlation with impact on prognosis? A retrospective study of 418 cases. Int. J. Clin. Exp. Med..

[71] Tao L., Xiang Y., Zeng X., Fu L., Li J., Chen H. (2024). Psychological-Distress Factors in Patients with Breast Cancer: A Qualitative Meta-Synthesis. J. Clin. Nurs..

[72] Andersen I.S., Jensen D.M.R., Grosen K., Bennedsgaard K.T., Ventzel L., Finnerup N.B. (2024). Body image and psychosocial effects in women after treatment of breast cancer: A prospective study. Am. J. Surg..

[73] Kissane D.W., Grabsch B., Clarke D.M., Smith G.C., Love A.W., Bloch S., Snyder R.D., Li Y. (2007). Supportive-expressive group therapy for women with metastatic breast cancer: Survival and psychosocial outcome from a randomized controlled trial. Psychooncology.

[74] Soto-Ruiz N., Escalada-Hernández P., Pimentel-Parra G.A., García-Vivar C. (2025). Quality of life in long-term cancer-free breast cancer survivors in Spain: A descriptive study. Sci. Rep..

[75] Ashton K., Oney K. (2024). Psychological Intervention and Breast Cancer. Curr. Breast Cancer Rep..

